# Safety of 810 nm photobiomodulation in the developing brain: no evidence of glial reactivity or pro-inflammatory cytokine expression in rats

**DOI:** 10.1007/s10103-025-04684-5

**Published:** 2025-10-06

**Authors:** Alba Gutiérrez-Menéndez, Lucía Rodríguez-Fernández, Candela Zorzo, Juan A. Martínez, Alina Diez-Solinska, Jorge L. Arias

**Affiliations:** 1 Universidad Internacional de Valencia (VIU), Valencia, Spain; 2 Instituto de Neurociencias del Principado de Asturias (INEUROPA), Oviedo, Spain; 3 Instituto de Investigación Sanitaria del Principado de Asturias (ISPA), Oviedo, Spain; 4 University of Oviedo, Oviedo, Spain; 5 Basque Country University, San Sebastian, Spain

**Keywords:** Photobiomodulation, Glial cells, Cytokines, Neuroinflammation, Developing brain, Near-infrared light

## Abstract

Photobiomodulation (PBM) is an innovative non-invasive light-based technique that uses wavelengths around red to infrared light to stimulate neural activity. Literature has addressed PBM’s effectiveness in healthy adult subjects, in several neurological conditions, and also in younger populations. However, there is still a lack of both preclinical and clinical studies that evaluate its safety during early developmental stages, when the brain is still maturing. We explored safety of PBM (810 nm) in young male Wistar rats by examining astrocytes and microglia cells thought GFAP and Iba1 immunohistochemistry, as well as the expression of pro-inflammatory cytokines (Interleukin-6, interleukin-1β and tumour necrosis factor α) through quantitative PCR, both in prefrontal cortex and hippocampus. Under the tested parameters and time point, PBM did not induce detectable glial reactivity or pro-inflammatory cytokine expression. This research highlights the potential use of 810 nm-PBM in the developing brain, providing preliminary evidence that this technique does not induce a neuroinflammatory response, representing an important first step to verifying the beneficial use of this technique without risks in paediatric and adolescence populations. More research is necessary to confirm the safety of PBM for different conditions and employing diverse parameters.

## Introduction

Photobiomodulation (PBM) therapy, involving non-invasive irradiation with low-power red to near-infrared (NIR) light (600–1000 nm), has emerged as a promising tool for modulating central nervous system (CNS) activity [1, 2]. Originally developed over five decades ago, PBM has demonstrated the capacity to neurostimulate or neuromodulate a variety of biological processes, becoming an innovative modality to treat a wide range of neurological conditions [3–5]. Indeed, the initial demonstration of PBM’s neurotherapeutic effect was achieved by Lapchak et al. (2004), who reported improved functional outcomes in an animal model of stroke following transcranial PBM application [6]. Interestingly, subsequent preclinical and clinical studies have shown that PBM can promote microcirculation, regeneration and cellular proliferation and can mitigates oxidative stress, oedema and inflammation in several medical conditions [4]. These findings support PBM as a non-invasive therapeutic modality with significant potential for neuromodulation and neuroprotection [7].

Although the precise mechanism of action of PBM is still being studied, it is known that the primary photoacceptor of red and near-infrared light is the cytochrome c oxidase (CCO) enzyme, also known as the complex IV of the mitochondrial respiratory chain [8]. The CCO plays a pivotal role in adenosine triphosphate (ATP) synthesis through oxidative phosphorylation. Also, upon photon absorption by CCO, mitochondrial membrane potential increases, electron transport is accelerated, and ATP production is enhanced, which leads to trigger the expression of genes involved in cell survival, proliferation, and tissue repair, among others [9]. Moreover, PBM can activate several signalling pathways, including those involved in neuroprotection, antioxidants, anti-inflammatory and anti-apoptosis pathways [4, 10]. Interestingly, it has also been show that PBM can upregulate neurotrophic factors such as brain-derived neurotrophic factor (BDNF) and nerve growth factor (NGF) and stimulate hippocampal neurogenesis, neuroplasticity and synaptic remodelling [10].

Due to the high energy demands of brain and its dependence on mitochondrial function, PBM therapy has been widely investigated as a potential treatment for a variety of neurological conditions [11]. Thus, preclinical and clinical research has demonstrated beneficial effects of PBM on cognitive function and related neural activity in both healthy adults and in patients suffering from several neurological conditions such as traumatic brain injury, Parkinson’s and Alzheimer’s disease and depression [12–14]. In addition, the use of this technique during early neurodevelopmental stages is being investigated [15]. Although the available evidence remains limited, recent clinical studies have demonstrated promising effects of PBM in children and adolescents with neurodevelopmental disorders, particularly autism spectrum disorder (ASD), attention-deficit/hyperactivity disorder (ADHD), and Down syndrome (DS), showing improvements in behavioral, cognitive, and attentional domains with minimal adverse effects [15–17]. In parallel, animal studies have evaluated PBM in prenatal and postnatal models, including neonatal hypoxia [18] and normal developmental contexts [19, 20], while others have studied its effect on healthy young or juvenile rat models [21–23]. However, current evidence is still limited by small sample sizes, lack of sham-controlled trials, high heterogeneity in stimulation parameters, and scarce mechanistic insight—particularly regarding long-term safety during brain maturation [15].

Given the growing interest in applying PBM in pediatric and adolescent clinical settings, particularly for neurodevelopmental disorders such as ASD and ADHD, it is critical to establish its safety during brain maturation. Preclinical studies are indispensable to provide objective evidence that PBM does not trigger adverse neuroinflammatory responses, thereby laying the foundation for its responsible translation into early-life clinical applications. Due to the aforementioned limited data on PBM safety during neurodevelopment, it is critical to examine potential neuroinflammatory responses that may arise from PBM interventions. Glial cells, particularly astrocytes and microglia, define brain homeostasis and act as first responders to physiological stress, injury, or infection [24]. Astrocytes are involved in neurotransmitter regulation, metabolic support, and maintenance of the blood–brain barrier [25]. Under pathological conditions they become reactive, altering their morphology and increasing the expression of glial fibrillary acidic protein (GFAP), a hallmark of astrogliosis [26]. On the other hand, microglia play a critical role as resident immunocompetent and phagocytic cells within the CNS. They are the resident immune cells of the CNS and constantly monitor the brain microenvironment. Upon detecting cellular distress or disruption of homeostasis, microglia undergo activation, characterized by changes in cell morphology, gene expression, and cytokine release [27, 28] Activated glia release pro-inflammatory cytokines such as interleukin-6 (IL-6), interleukin-1β (IL-1β), and tumor necrosis factor-alpha (TNF-α), which orchestrate the brain’s innate immune response [29, 30] While these mechanisms are essential for defending the CNS, sustained or inappropriate activation may result in collateral damage to neural circuits and impair neurodevelopmental trajectories.

Despite the increasing clinical application of PBM in pediatric and adolescent populations, there is a striking lack of preclinical evidence assessing its safety during early neurodevelopmental stages. In particular, the potential of PBM to induce glial reactivity or trigger neuroinflammatory processes in the developing brain remains largely unexplored. Addressing this gap is essential to ensure the responsible translation of PBM into pediatric clinical practice. In light of these considerations, the present study aimed to evaluate the neuroinflammatory safety profile of transcranial PBM in juvenile rats during a critical period of brain development. Specifically, we investigated glial reactivity and cytokine expression in healthy male Wistar rats (postnatal day (PND) 29) following exposure to an 810 nm NIR wavelength. Immunohistochemical analyses were performed to assess astrogliosis and microglial activation in the prefrontal cortex (PFC) and hippocampus (HPC), using glial fibrillary acidic protein (GFAP) and ionized calcium-binding adapter molecule 1 (Iba1) as respective markers. Additionally, we examined mRNA expression of pro-inflammatory cytokines Interleukin-6 (IL-6), interleukin-1β (IL-1β) and the tumour necrosis factor α, (TNFα), by quantitative polymerase-chain-reaction (PCR), in the same brain regions.

## Methods

### Animals

Forty-two male Wistar rats aged between 23 and 29 days old from eight different litters were used. The animals were housed in groups in polycarbonate cages located at a constant temperature (22 ± 2 °C) with a relative humidity of 65–70% and a 12-h artificial light (350 luxes)-dark cycle (8:00–20:00/20:00–8:00). They had *ad libitum* access to food and tap water.

The animals were randomly divided into three different groups for the study of glial density: PBM group (PBM, *n* = 8), which received the PBM administration; SHAM group (SHAM; *n* = 8), which underwent the same procedures as the PBM group but the device was switched off, and a control group (C; *n* = 8), kept in their cages.

The remaining animals were randomly split into two groups to study the pro-inflammatory cytokines: PBM group (PBM, *n* = 8) and SHAM group (SHAM, *n* = 10).

All animal procedures were carried out accordance with Directive 2010/63/EU of the Council of the European Communities and Royal Decree No 53/2013 of the Ministry of the Presidency on the protection of animals used for experimentation and other scientific purposes. The Ethics Committee of the Principality of Asturias approved the study.

## Photobiomodulation administration

The PBM procedure used is based on our previous research and is described in Gutiérrez-Menéndez et al. (2022) [23]. Briefly, PBM and SHAM groups were subjected to the procedure for 7 days. Habituation to the procedure was carried out on the first 2 days (PND 22 and PND 23): the first third of the animal’s heads were shaved, just between the eyes, trying to maximize light penetration through the prefrontal areas and then, the animals were immobilized by the researcher on a soft surface and the switched-off PBM was placed over the shaved region for 30 min. During the following 5 days (PND 24-PND 28), the same immobilised procedure was carried out, but the PBM device was in ON mode for the PBM groups and remained OFF for the SHAM groups. The laser used was a continuous wave at 810 nm wavelength, operated at an output power of 40 mW and irradiance of 65.6 W/m^2^, with a beam size of 0.0495 cm^2^. PBM groups received 36 cycles of PBM (40 s ON and 10 s OFF), reaching a total irradiation time of 24 min and an average fluence of 46.5 J/cm^2^ per day. Approximately 0.8% of the applied power reaches the brain tissue, previously determined in rat skulls using a PM 160 optical power meter (ThorLabs, United States).

### Brain processing

On PND 29, in order to analyse the glial density of astrocytes and microglia using a GFAP-IR and an Iba1-IR immunostaining, respectively, animals from the three designated groups (PBM, SHAM and C) were anaesthetised with ketamine (80 mg/kg) and xylazine (10 mg/kg) and perfused transcardially with 0.9% saline for 5 min followed by 4% paraformaldehyde phosphate buffer (0.1 M; pH 7.4) for 20 min. Brains were removed and post-fixed in 4% paraformaldehyde (0.1 M; pH 7.4) overnight and with 30% sucrose phosphate buffer (0.1 M; pH 7.4) until they sank. Brains were then dehydrated and embedded in paraffin. Coronal Sect. 30-µm thick of the brain were sliced using a microtome (Leica, RM2135, Germany). Two series of adjacent sections were obtained, one for GFAP-IR immunocytochemistry and the other for Iba1 IR immunocytochemistry. Regions of interest were anatomically defined according to Paxinos and Watson’s atlas (2007) [31], and the distance in mm of the regions counted from bregma was: +3.20 mm for the cingulate (CG), prelimbic (PL) and infralimbic (IL) cortices and − 2.28 mm for CA1, CA3 and dentate gyrus (DG) subfields of the dorsal hippocampus.

To study pro-inflammatory cytokines, animals from the PBM and SHAM groups were decapitated, brains were removed, PFC and HPC were isolated following the procedure described by Spijker (2011) [32] and frozen rapidly in isopentane. In particular, the PFC was isolated from rostral frontal cortices encompassing cingulate, prelimbic and infralimbic areas, using the genu of the corpus callosum as landmarks and the overlying motor cortices were excluded. The HPC was dissected by gently separating the hippocampal formation from surrounding cortex along the lateral ventricle and fimbria, and the adjacent cortical tissue were carefully removed. Tissue was stored at − 80 °C to measure relative mRNA relative expression of IL-6, IL-1β and TNFɑ.

### GFAP-IR and Iba1-IR immunocytochemistry

The protocol followed was previously described by Banqueri et al. (2019) [33]. Briefly, sections of perfused tissue were deparaffinised, hydrated, washed in Tris buffer saline (TBS) containing 0.1% Triton (TBS-T0.1%) and blocked with 1% human serum (SIGMA A-1653) in TBS. Then, washed with TBS-T0.1% and incubated overnight with a polyclonal primary antibody rabbit anti-GFAP (1:800; Dako, Denmark) or rabbit anti-Iba1 (1:480; Fujifilm Wako chemicals Europe). Next, sections were dipped in TBS-T0.1%, incubated with biotinylated secondary goat anti-rabbit IgG antibody (1:480; Pierce, USA) and washed with TBS-T0.1%. Afterward, sections were incubated with an avidin–biotin peroxidase complex (Vectastain ABC Ultrasensitive Elite Kit, Thermo Fisher Scientific, Inc.) and washed in TBS-T0.1% and TBS. Then, the reaction was visualised by treating the sections in a solution of TBS, 0.05% w/v diaminobenzidine tetra-hydrochloride (Sigma-Aldrich, Madrid, Spain) and 33% (v/v) hydrogen peroxidase solution. Finally, the tissue was dehydrated, cleared with xylene and coverslipped with Entellan (Merck, Germany). All the immunohistochemistry procedures included sections that served as controls where the primary antibody was not added.

### GFAP and Iba1 quantification

**Fig. 1:**
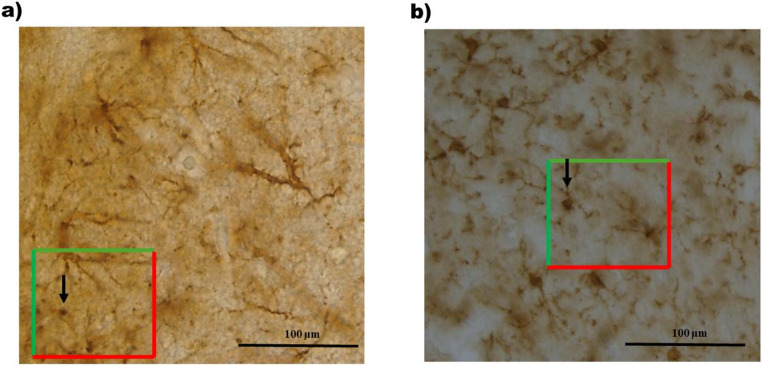
Representative images of GFAP-IR (**a**) and Iba1-IR (**b**) cells acquired using a microscope (Leica DFC490, Germany) coupled to a computer with Leica Application Suite X software (Leica Microsystems, Germany), with a 20x objective and a systematic random sampling strategy. Each image shows a counting frame (disector) superimposed on the region of interest. Arrows indicate immunopositive cells with clearly defined nuclei within the disector area that were included in the quantification. Cells touching the upper or left borders (green borders) were included, while those contacting the lower or right borders (red borders) were excluded, in accordance with the standard inclusion/exclusion criteria. Scale bar: 100 μm. µm2: square micrometer

The number of GFAP-IR and Iba1-IR cells was quantified in three alternating sections with a constant distance between them (90 μm, to avoid double counting of cells) using a microscope (Leica DFC490, Germany) coupled to a computer with Leica Application Suite X software (Leica Microsystems, Germany) and with a numerical aperture 20x objective. Within each section, quantification was carried out using a systematic random sampling strategy, in which predefined counting frames were superimposed across each region of interest. GFAP-IR and Iba1-IR cells were counted only if they had a clearly defined nucleus within the disector area. For cells located at the frame borders, a conventional inclusion-exclusion criterion was applied: nuclei touching the upper or left borders were included, whereas those contacting the lower or right borders were excluded, to prevent double counting (Fig. 1). Frames sizes for both GFAP-IR and Iba1-IR cells were 200 × 200 µm^2^ for CG, PL, and IL; 150 × 200 µm^2^ for CA1; 150 × 80 µm^2^ for CA3 and 100 × 100 µm^2^ for DG. A total of twelve disectors per subject were used in CG, IL and PL and nine in CA1, CA3 and DG. Results show immunopositive cells’ mean x 1000/area of quantification (µm^2^).

### Real-time PCR analysis of mRNA cytokine expression

The total RNA of each structure was isolated using the NucleoSpin RNA Plus kit (Macherey Nagel, Germany). A UV spectrophotometric analysis was performed at 260 nm to determine the RNA concentrations, while the 260:280 absorbance ratio was utilised to assess the nucleic acid purity (Epoch, BioTek Instruments, Inc., Winooski, VT, USA). The total RNA was then reverse transcribed with the PrimeScript RT reagent kit (Takara Bio Inc., Madrid, Spain). The resulting cDNA levels were quantified by SYBR Green-based (SYBR^®^Premix Ex TaqTM, Takara Bio Inc., Madrid, Spain) real-time PCR, and the formation of PCR products was monitored using the 7500 Real-Time PCR System (Applied Biosystems, Madrid, Spain). The cDNA sequences were obtained from the Ensenbl repository (https://www.ensembl.org/index.html). Glyceraldehyde-6-phosphate dehydrogenase (GAPDH) and hypoxanthine phosphoribosyltransferase 1 (HPRT1) were used as the housekeeping genes. The primer sequences were designed using Primer3web version 4.1.0 (https://primer3.ut.ee/) and obtained from Metabion International AG. Prime specificity was verified by melt curve analysis. The relative gene expression was determined using the 2^−∆∆Ct method [34], with the mean Ct value of the housekeeping genes serving as the normalization reference. Primer sequences used for PCR were as follows (primer sequence direction is 5′ − 3′): IL-6, forward: CTGCTCTGGTCTTCTGGAGT, reverse: TGTTGCTCAGATCTCCCTTCT; IL-1β, forward: ATCTCACAGCAGCATCTCGA, reverse: AGGACGGGCTCTTCTTCAAA; TNFα, forward: CAGACCCTCACACTCAGATCA, reverse: AGCCTTGTCCCTTGAAGAGA.

### Statistical analysis

All data were analysed and graphically represented using Sigma-Stat 12.5 software (Systat, Richmond, USA) (data are expressed as mean ± standard error of the mean, SEM). Differences were considered statistically significant when *p* < 0.05. The Shapiro-Wilk test was used to test the assumption of normality (*p* > 0.05) and Levene’s test for equality of variances (*p* > 0.05). When data fit both assumptions, one-way ANOVA was used, whereas Kruskal-Wallis one-way analysis of variance on ranks was performed when equality of variances was not met. No post hoc tests were performed, as no statistically significant differences were observed.

## Results

**Fig. 2:**
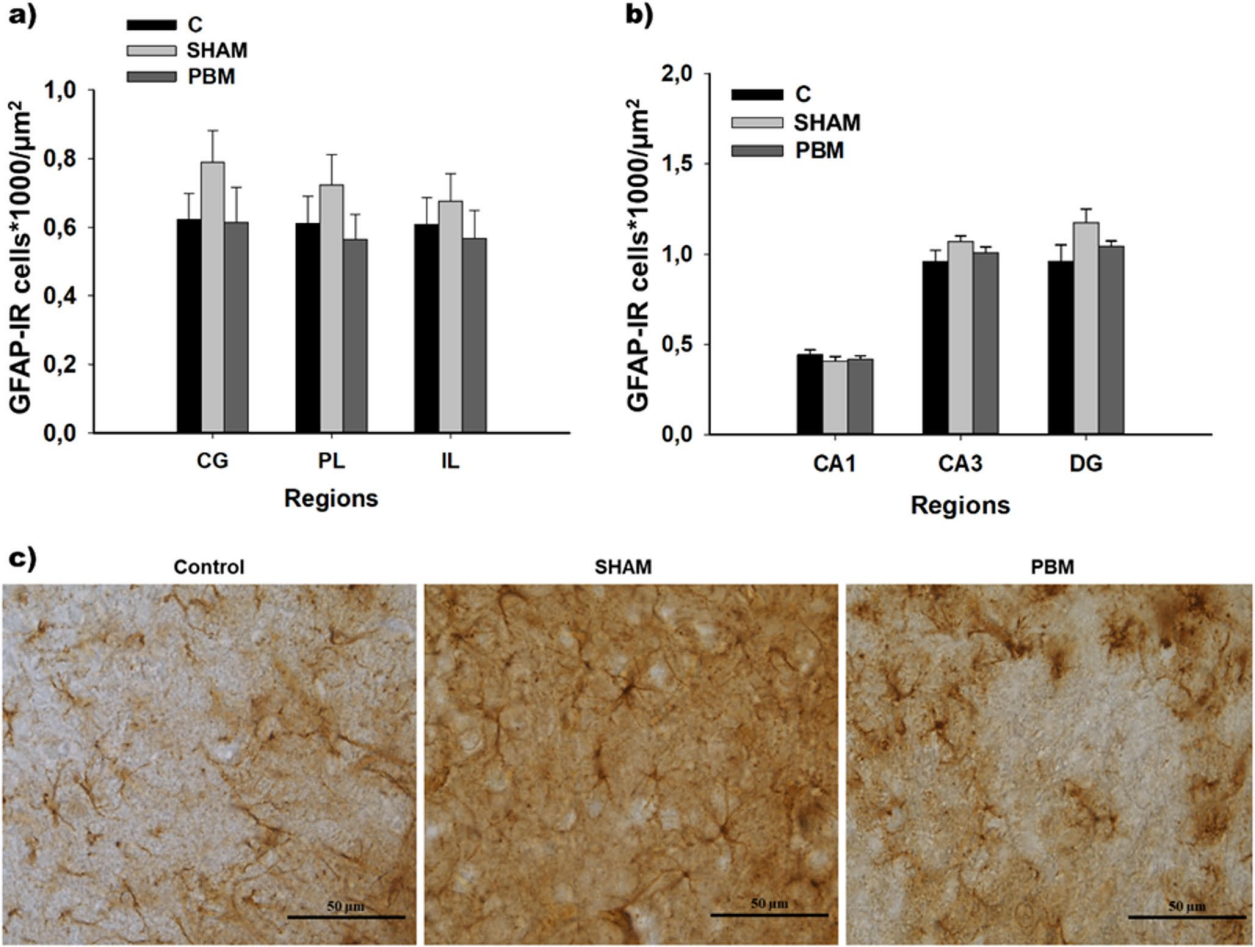
Astrocytic cell density (mean ± SEM). There were no differences in the number of GFAP-IR cells*1000/µm^2^ between groups in the prefrontal cortex (CG, PL and IL) (**a**) or the hippocampus (CA1, CA3 and DG) (**b**) (*p* > 0.05). Statistical analysis was performed using one-way ANOVA for CG (C, *n =* 6; SHAM *n* = 6, PBM *n* = 6), PL (C, *n =* 6; SHAM, *n* = 6; PBM, *n* = 6), IL (C, *n =* 6; SHAM, *n* = 6; PBM *n* = 6), CA1 (C, *n =* 7; SHAM *n* = 6; PBM, *n* = 8) and CA3 (C, *n =* 7; SHAM, *n* = 6; PBM, *n* = 8), and Kruskal-Wallis one-way analysis of variance on ranks for DG (C, *n =* 7; SHAM, *n* = 6; PBM, *n* = 8). (**c**) Representative images of GFAP-IR cells for each experimental group (control, sham and PBM group). The images were acquired using a 40x objective. Scale bar: 50 μm. Groups: C, control group; SHAM group, with the PBM switched off; PBM, photobiomodulation group. Areas: CG, Cingulate cortex; PL, Prelimbic cortex; IL, Infralimbic cortex; CA1, field CA1 of hippocampus; CA3, field CA3 of hippocampus; DG, Dentate Gyrus. GFAP-IR: glial fibrillary acidic protein immunoreactive cells; µm2: square micrometer

A one-way ANOVA was carried out to analyse glial density between groups in the PFC and HPC. Under the tested parameters and time point, no differences were found in the density of GFAP-IR cells in the subregions of the PFC (CG: F_(2,15)_ = 1.163, *p* = 0.339; PL: F_(2,15)_ = 1.084, *p* = 0.375; IL: F_(2,15)_ = 0.470, *p* = 0.637) (Fig. 2a) or in the HPC (CA1: F_(2,18)_ = 0.588, *p* = 0.566; CA3: F_(2,18)_ = 1.474, *p* = 0.255; DG: H_*2*_ = 2.334, *p* = 0.311) (Fig. 2b).

**Fig. 3:**
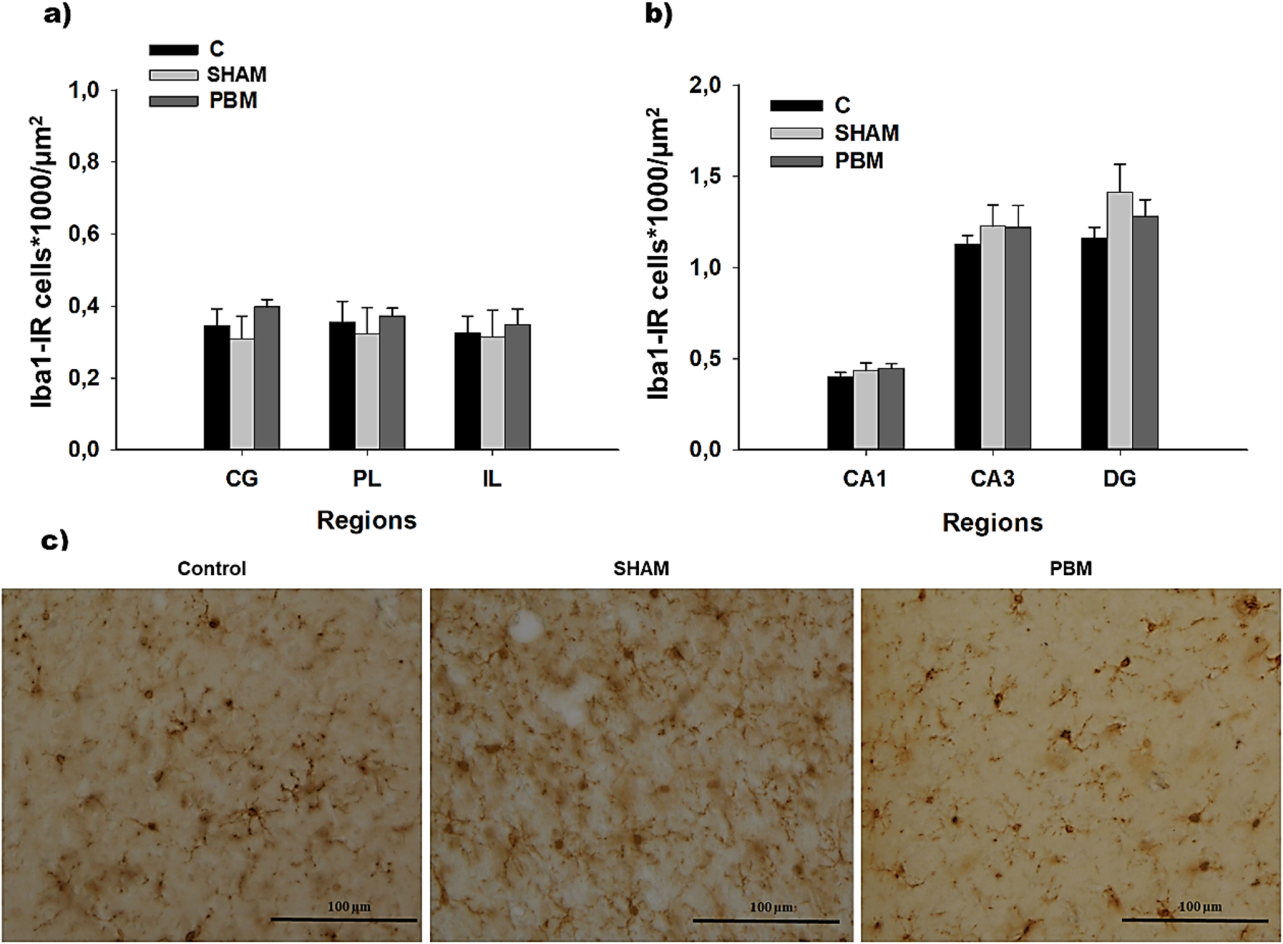
Microglial cell density (mean ± SEM). No differences in the number of Iba1-IR cells*1000/µm2 were found in the prefrontal cortex (CG, PL and IL) (**a**) or the hippocampus (CA1, CA3 and DG) (**b**) (*p* > 0.05). Statistical analysis was performed using one-way ANOVA for all regions (CG: C, *n =* 6; SHAM, *n* = 6; PBM *n* = 6; PL: C, *n =* 6; SHAM, *n* = 6; PBM, *n* = 6; IL: C, *n =* 6; SHAM, *n* = 6; PBM, *n* = 6; CA1: C, *n =* 8; SHAM, *n* = 6; PBM, *n* = 8; CA3: C, *n =* 8, SHAM, *n* = 6; PBM, *n* = 8 and DG: C, *n =* 8; SHAM, *n* = 6; PBM, *n* = 8). (**c**) Representative images of Iba1-IR cells for each experimental group (control, sham and PBM group). The images were acquired using a 40x objective. Scale bar: 100 μm. Groups: C, control group; SHAM group, with the PBM switched off; PBM, photobiomodulation group. Areas: CG, Cingulate cortex; PL, Prelimbic cortex; IL, Infralimbic cortex; CA1, field CA1 of hippocampus; CA3, field CA3 of hippocampus; DG, Dentate Gyrus. Iba1-IR: ionized calcium-binding adapter molecule 1 immunoreactive cells; µm^2^: square micrometer

Similarly, under the tested conditions, no differences were found in Iba1-IR cell density between groups in the PFC (CG: F_(2,15)_ = 0.932, *p* = 0.415; PL: F_(2,15)_ = 0.200, *p* = 0.821; IL: F_(2,15)_ = 0.101, *p* = 0.904) (Fig. 3a) or in the HPC (CA1: F_(2,19)_ = 0.795, *p* = 0.466; CA3: F_(2,19)_ = 0.337, *p* = 0.718; DG: _(2,19)_ = 1.512, *p* = 0.246) (Fig. 3b).

**Fig. 4:**
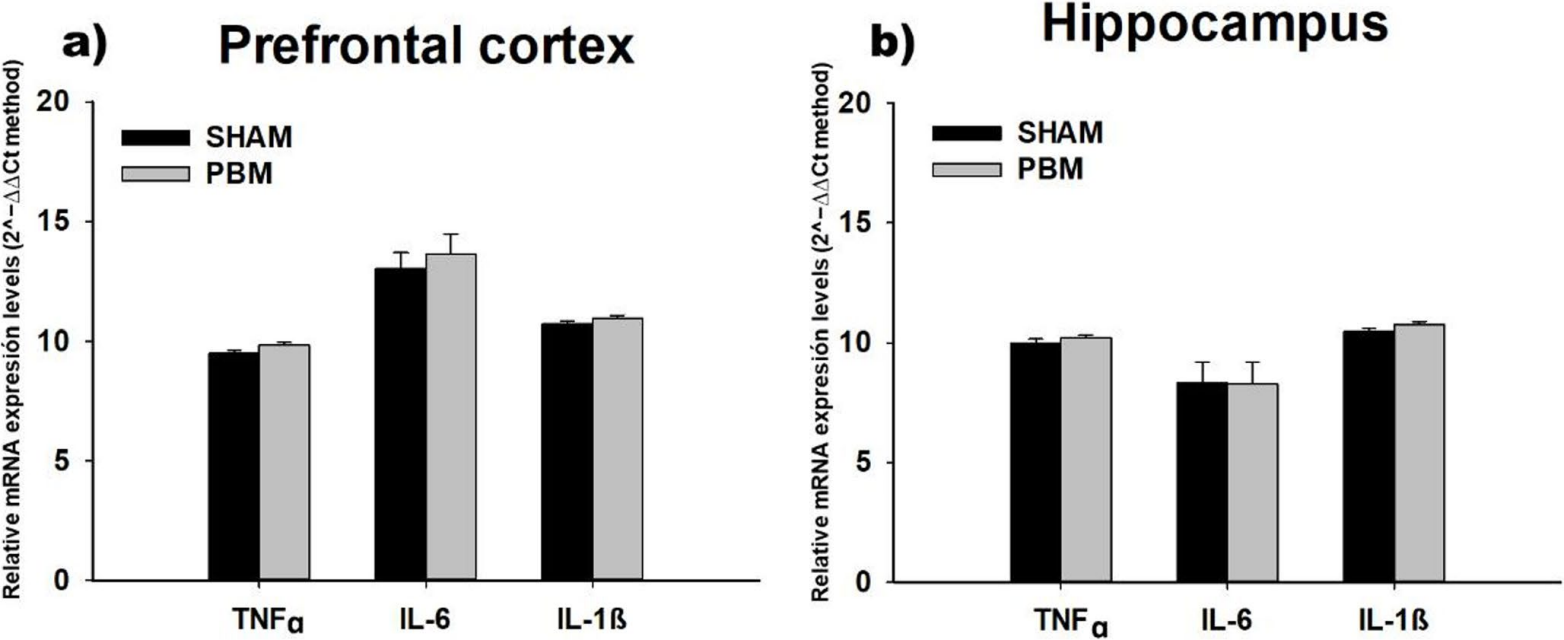
Relative expression levels of pro-inflammatory cytokines in the prefrontal cortex (PFC) (**a**) and the hippocampus (HPC) (**b**) of the SHAM and PBM groups (mean ± SEM). No significant differences were found in relative TNFɑ, IL-6 or IL-1β mRNA expression between groups in either region (*p* > 0.05). Statistical analysis was performed using one-way ANOVA for TNFɑ (SHAM, *n* = 10; PBM, *n* = 8), IL-6 (SHAM, *n* = 3; PBM, *n* = 3) and IL-1β (SHAM, *n* = 10; PBM, *n* = 8) in the prefrontal cortex and for TNFɑ (SHAM, *n* = 10; PBM, *n* = 8) and IL-1β (SHAM, *n* = 10; PBM, *n* = 8) in the hippocampus, and Kruskal-Wallis one-way analysis of variance on ranks for IL-6 (SHAM, *n* = 8; PBM, *n* = 8) in the hippocampus. Groups: SHAM group, with the PBM switched off; PBM, photobiomodulation group TNFα, tumour necrosis factor α; IL-6, interleukin-6; IL-1β: interleukin-1β; mRNA: messenger RNA; 2^−∆∆Ct method: relative quantification of gene expression

Regarding cytokine analysis, no differences were found between the SHAM and PBM groups in the relative TNFɑ, IL-6 and IL-1β RNA expression levels in the PFC (TNFɑ: F_(1,16)_ = 3.827, *p* = 0.068; IL-6: F_(1,4)_ = 0.317, *p* = 0.604; IL-1β: F_(1,16)_ = 2.484, *p* = 0.135) or in the HPC (TNFɑ: F_(1,16)_ = 0.820, *p* = 0.379; IL-6: H_1_ = 0.099, *p* = 0.798; IL-1β: F_(1,16)_ = 2.365, *p* = 0.144), at the examined time point (Fig. 4).

## Discussion

This study investigated the neuroinflammatory safety profile of transcranial PBM in healthy juvenile male rats following five consecutive days of near-infrared (810 nm) light exposure. Under the tested parameters and time point, we did not find changes in astrocytic or microglial density in any of the immunostained regions, nor in the determination of cytokine mRNA expression (IL-6, IL-1β and TNFα) in the PFC and the HPC. These results suggest that PBM, applied under the tested parameters, does not elicit neuroinflammatory responses in the developing brain, supporting its safety during early postnatal stages.

PBM delivers non-invasive red/NIR light to biological tissues to achieve therapeutic advantages, restore function, or enhance physiological processes [8]. A large body of literature focuses on using PBM to improve cognitive functions in healthy adults [35], or as a therapy for diverse neurological disorders [5, 11, 36–38] mostly in adult samples, including elderly [39, 40]. However, the optimal parameters for PBM therapy in the brain—including wavelength, fluence, power density, number of sessions, and duration—are still under investigation, and no clear consensus has been reached [3]. As a result, outcomes remain variable, and clinical applications continue to be debated [14]. Regarding safety, the low power density irradiation seems insufficient to cause thermal damage, and the preclinical [41] and clinical trials have not reported major adverse events [14]. Nonetheless, some studies have described mild side effects in human participants, such as diastolic blood pressure increases [42], headaches, irritability, or the perception of vivid colours [43]. These adverse effects seem to depend on the choice of optical parameters due to light penetration in the tissue depending on them [3]. Interestingly, recent analyses suggest that individual variations in skull bone density and thickness have minimal impact on light penetration at 810 nm, further supporting its clinical applicability [44].

PBM has been used not only in adult samples but also in young subjects suffering from several pathologies, although the studies are limited. In the preclinical field, the application of 808 nm-PBM on a neonatal hypoxic ischemia rat model alleviated cortex-related motor deficit and hippocampus-related memory and learning dysfunction, along with the protection of the mitochondrial function and integrity [18]. Also, recent research which applied 830 nm-PBM to 56 days-old mice exposed to valproic acid as a model of ASD, observed that the therapy decreased pathological ASD behaviours along with attenuation of glial activation [45]. In human studies, PBM research has also mainly focused on the treatment of ASD in children and adolescents. Thus, it has administered 635 nm- PBM to children and adolescents (5–17 years) for 4 weeks, finding a reduction of irritability and other symptoms and behaviours associated with the ASD [16]. Similarly, Pallanti et al. (2022) [17] also detected a decrease in ASD severity, noncompliant behavior and parental stress, and cognitive rigidity after applying 810 nm- PBM to children and adolescents (5–15 years). Beyond ASD, recent clinical studies have extended the application of PBM to other neurodevelopmental disorders, ADHD and DS, yielding preliminary encouraging results. Improvements have been observed in attentional control, global functioning, and verbal fluency, with minimal adverse effects reported across studies. These findings, together with evidence from ASD interventions, suggest that PBM may exert beneficial effects on core cognitive and behavioral domains commonly impaired in neurodevelopmental disorders [15]. Interestingly, the proposed mechanisms include enhanced mitochondrial respiration, reduced oxidative stress, and modulation of neuroinflammatory markers, which are pathophysiological features frequently shared across some neurodevelopmental disorders.

Beyond pathological models, PBM has also been explored in healthy animals at different developmental stages to investigate its potential as a metabolic or neuroplastic enhancer [46]. Preclinical research in this field has focused on embryonic development, the neonatal stage, and the postnatal/juvenile period. Thus, Buzzá et al. (2018) [19] used 630 nm-red light to radiate the formation of healthy chicken eggs, and found higher efficiency in embryo development. Subsequently, in 2019, using the same radiation in healthy rat pups (newborns), they detected faster eye-opening and weight gain by irradiating puppy bodies [20]. In addition, this technique has also been used in healthy young subjects. In this case, enhancement of the metabolic pathway has been reported, particularly for excitatory neurotransmission and oxidative metabolism in 4-month-old rats, and no metabolic and c-Fos positive changes in 29-day old rats after 810 nm-PBM [21, 23]. Taken together, these findings highlight the heterogeneous effects of PBM in the developing brain and reinforce the need for studies specifically addressing its safety in healthy juvenile populations.

In our study, five sessions of transcranial PBM (810 nm) applied to healthy juvenile rats (29 PND) did not induce changes in astrocytic or microglial cell density in the PFC or HPC. Astrocytes which play a central role in maintaining CNS homeostasis, typically become reactive in response to injury or inflammation, a process characterized by hypertrophy and overexpression of GFAP [25]. Along the same line, microglia, the innate immune cells of the CNS, also respond rapidly to pathological stimuli through changes in morphology and upregulation of Iba1 expression [28, 47]. Therefore, pathological brain conditions induce reactive astrocytes and microglia, resulting in neuroinflammation [26]. The absence of cellular changes in these glial populations in our study suggests that, under the tested conditions, PBM did not trigger gliosis or immune activation in non-pathological juvenile brains.

To further assess whether PBM induces subtle inflammatory signalling, we also evaluated mRNA expression of the pro-inflammatory cytokines IL-6, IL-1β and TNFα in the HPC and PFC. Consistent with the glial findings, no significant group differences were observed in cytokine expression levels at the examined time point. To our knowledge, this is one of the few preclinical studies to assess the expression of pro-inflammatory cytokines (IL‑6, IL‑1β, and TNFα) via PCR in the developing brain following PBM, and it is important to outline that this molecular approach allows for the detection of subtle inflammatory responses that may not be evident at the histological level, providing a more sensitive assessment of the safety profile of PBM when applied during early neurodevelopment. In pathological conditions, several studies have reported that PBM exerts anti-inflammatory effects. A recent systematic review [48] summarized evidence from animal models of neurological diseases (ages ranging from 7 weeks to 20 months) and showed that PBM can reduce pro-inflammatory mediators while enhancing anti-inflammatory signaling. Similarly, El Massri et al. (2018) [49] reported attenuation of glial activation in aged animals after 670 nm-PBM, and Cardoso et al. (2022) [48] suggested that 660 nm-PBM improves inflammatory responses and vascular signaling pathways in the aged brain. In young pathological models, Kim et al. (2022) [45] demonstrated reduced astrocytic and microglial activation in a prenatal valproic acid model of ASD, while Yang et al. (2021) [45] found decreased neuroinflammation after 808 nm-PBM in neonatal hypoxic-ischemic rats. Together, these findings support the notion that PBM around 800 nm can modulate neuroinflammation in vulnerable brains. Our study complements this literature by showing that, under physiological conditions in the developing brain, repeated 810 nm PBM did not elicit detectable glial or cytokine responses, thus providing preliminary safety evidence.

It is important to consider the broader limitations identified in the current literature. Most available studies involve small sample sizes and lack rigorous methodological controls such as randomization, sham stimulation, or blinded assessment. Moreover, stimulation protocols vary widely in terms of wavelength, pulse frequency, intensity, and target location, which limits reproducibility and cross-study comparison. Only a few trials incorporate objective biomarkers, such as neuroimaging, EEG, or cytokine assays, to elucidate the underlying mechanisms or treatment response profiles [15]. It should be noted that the physiological effects of PBM depend not only on stimulation parameters but also on the biological characteristics of the subject receiving the treatment. Age, brain maturation, and tissue composition can modulate how light interacts with neural tissue, making it essential to evaluate PBM specifically in the developing brain. Thus, our research provides evidence of the safe use of the 810 nm-PBM in young healthy subjects regarding neuroinflammation. This wavelength belongs to the near-infrared (NIR) therapeutic window (700–1000 nm), known for its optimal tissue penetration due to minimal absorption by water and hemoglobin [50], with 810 nm standing out as one of the most effective for transcranial applications [51]. This wavelength has been shown to stimulate CCO, enhancing mitochondrial activity and ATP production [52], although it has been shown that a combination of 660 and 810 nm also increases CCO activity in both male and female rats [53]. In the same line, Sanderson et al. (2018) [50] confirmed 810 nm-PBM is a photostimulatory wavelength that had a stimulating CCO activity while 750 and 950 nm suppressed oxygen consumption by the CCO, suggesting a wavelength-dependent dual effect. These wavelength-specific responses reinforce the need for careful parameter selection and underscore the importance of evaluating PBM effects in specific biological contexts. Taken together, our findings contribute to establishing a safety framework for PBM applications during early life stages and emphasize the importance of combining histological and molecular markers to evaluate subtle immune activation in neurodevelopmental settings.

Some limitations of the present study should be acknowledged. First, analyses were conducted at a single developmental time point (PND 29), which does not capture potential longer-term effects. Second, only male rats were included, so sex-dependent responses remain to be explored. Third, the sample size was relatively modest, although comparable to other preclinical PBM studies. Finally, we restricted our outcome measures to glial cell density and cytokine mRNA levels. While these are widely used and sensitive markers of neuroinflammation, complementary approaches such as protein quantification (Western blot/ELISA), immunofluorescence analysis of glial reactivity, or long-term follow-up assessments would provide additional insights. Additionally, the absence of a non-handled control group in the qPCR analysis may limit the interpretability of the results. Future studies should therefore aim to include different developmental stages, both sexes, non-handled control group, additional molecular and protein markers, and longitudinal designs to more comprehensively characterize the safety of PBM across molecular, cellular, and temporal dimensions.

## Conclusions

Under the tested parameters and time point, the application of PBM at 810-nm for 5 consecutive days in healthy young male rats (29 PND at the end of the experiment) did not generate changes in astrocytic or microglial density in any of the immunostained regions nor in the determination of cytokine mRNA expression (IL-6, IL-1β and TNFα) in the PFC and the HPC. These findings provide initial evidence supporting the safety of PBM in the developing brain and suggest that this technique may be applied without triggering neuroinflammatory responses under physiological conditions. This study represents a first step toward validating the safe use of PBM in pediatric populations; however, further research is required to confirm its safety across different developmental stages, clinical conditions, and stimulation protocols.

## Data Availability

No datasets were generated or analysed during the current study.

## Abbreviations

ASD: autism spectrum disorder
ATP: adenosine triphosphate, BDNF: brain-derived neurotrophic factor
CCO: cytochrome c oxidase
CG: cingulate cortex
CNS: central nervous system
DG: dentate gyrus
GAPDH: glyceraldehyde-6-phosphate dehydrogenase
GFAP: glial fibrillary acid protein
GFAP-IR: glial fibrillary acidic protein immunoreactive cells
HPC: hippocampus
HPRT1: hypoxanthine phosphoribosyltransferase 1
Iba1: ionized calcium-binding adapter molecule 1
Iba1-IR: ionized calcium-binding adapter molecule 1 immunoreactive cells
IL-6: interleukin-6
IL-1β: interleukin-1β
IF: infralimbic cortex
NGF: nerve growth factor
NIR: near-infrared light
PBM: photobiomodulation
PFC: prefrontal cortex
PL: prelimbic cortex
PND: postnatal day
TBS: tris buffer saline
SEM: standard error of the mean
TBS-T: tris buffer saline triton
TNFα: tumour necrosis factor α

## References

[1] Zhao C, Li D, Kong Y et al (2022) Transcranial photobiomodulation enhances visual working memory capacity in humans. Sci Adv 8:1–12 10.1126/sciadv.abq3211 PMC10936045 36459562

[2] Lee Tlok, Ding Z, Chan AS (2023) Can transcranial photobiomodulation improve cognitive function? A systematic review of human studies. Ageing Res Rev 83:101786. 10.1016/j.arr.2022.101786 36371017 10.1016/j.arr.2022.101786

[3] Salehpour F, Mahmoudi J, Kamari F et al (2018) Brain photobiomodulation therapy: a narrative review. Mol Neurobiol 55:6601–6636. 10.1007/s12035-017-0852-4 29327206 10.1007/s12035-017-0852-4 PMC6041198

[4] Salehpour F, Khademi M, Hamblin MR (2021) Photobiomodulation therapy for dementia: a systematic review of pre-clinical and clinical studies. J Alzheimers Dis 83:1431–1452 33935090 10.3233/JAD-210029

[5] Shen Q, Guo H, Yan Y (2024) Photobiomodulation for neurodegenerative diseases: a scoping review. Int J Mol Sci. 10.3390/ijms25031625 38338901 10.3390/ijms25031625 PMC10855709

[6] Lapchak PA, Wei J, Zivin JA (2004) Transcranial infrared laser therapy improves clinical eating scores after embolic strokes in rabbits. Stroke 35:1985–1988. 10.1161/01.STR.0000131808.69640.b7 15155955 10.1161/01.STR.0000131808.69640.b7

[7] Cardoso FDS, de Souza Oliveira Tavares C, Araujo BHS et al (2021) Improved Spatial Memory And Neuroinflammatory Profile Changes in Aged Rats Submitted to Photobiomodulation Therapy. Cell Mol Neurobiol 1–12. 10.1007/s10571-021-01069-4 10.1007/s10571-021-01069-4 PMC11421705 33704604

[8] Stevens AR, Hadis M, Milward M et al (2023) Photobiomodulation in acute traumatic brain injury: a systematic review and meta-analysis. J Neurotrauma 40:210–227. 10.1089/neu.2022.0140 35698294 10.1089/neu.2022.0140

[9] Ramezani F, Neshasteh-Riz A, Ghadaksaz A et al (2022) Mechanistic aspects of photobiomodulation therapy in the nervous system. Lasers Med Sci 37:11–18. 10.1007/s10103-021-03277-2 33624187 10.1007/s10103-021-03277-2

[10] Xuan W, Agrawal T, Huang L et al (2015) Low-level laser therapy for traumatic brain injury in mice increases brain derived neurotrophic factor (BDNF) and synaptogenesis. J Biophotonics 8:502–511. 10.1002/jbio.201400069 25196192 10.1002/jbio.201400069 PMC5379854

[11] Montazeri K, Farhadi M, Fekrazad R et al (2021) Transcranial photobiomodulation in the management of brain disorders. Journal of Photochemistry and Photobiology B: Biology 221:112207. 10.1016/j.jphotobiol.2021.112207 34119804 10.1016/j.jphotobiol.2021.112207

[12] Gutiérrez-Menéndez A, Marcos-Nistal M, Méndez M, Arias JL (2020) Photobiomodulation as a promising new tool in the management of psychological disorders: a systematic review. Neurosci Biobehav Rev 119:242–254. 10.1016/j.neubiorev.2020.10.002 33069687 10.1016/j.neubiorev.2020.10.002

[13] El Massri N, Lemgruber AP, Rowe IJ et al (2017) Photobiomodulation-induced changes in a monkey model of parkinson’s disease: changes in tyrosine hydroxylase cells and GDNF expression in the striatum. Exp Brain Res 235:1861–1874. 10.1007/s00221-017-4937-0 28299414 10.1007/s00221-017-4937-0

[14] Yang M, Yang Z, Wang P, Sun Z (2021) Current application and future directions of photobiomodulation in central nervous diseases. Neural Regen Res 16:1177–1185. 10.4103/1673-5374.300486 33269767 10.4103/1673-5374.300486 PMC8224127

[15] Coelho DRA, Renet C, López-Rodríguez S et al (2024) Transcranial photobiomodulation for neurodevelopmental disorders: a narrative review. Photochem Photobiol Sci Off J Eur Photochem Assoc Eur Soc Photobiol 23:1609–1623. 10.1007/s43630-024-00613-7 10.1007/s43630-024-00613-7 39009808

[16] Leisman G, Machado C, Machado Y, Chinchilla-Acosta M (2018) Effects of Low-Level laser therapy in autism spectrum disorder. Adv Exp Med Biol 1116:111–130. 10.1007/5584_2018_234 29956199 10.1007/5584_2018_234

[17] Pallanti S, Di Ponzio M, Grassi E et al (2022) Transcranial photobiomodulation for the treatment of children with autism spectrum disorder (ASD): a retrospective study. Children (Basel) 9:755. 10.3390/children9050755 35626932 10.3390/children9050755 PMC9139753

[18] Yang L, Dong Y, Wu C et al (2021) Effects of prenatal photobiomodulation treatment on neonatal hypoxic ischemia in rat offspring. Theranostics 11:1269–1294. 10.7150/thno.49672 33391534 10.7150/thno.49672 PMC7738878

[19] Buzzá HH, Zangirolami AC, Kurachi C, Salvador V (2018) Photostimulation effects on chicken eggs development: perspectives to human newborns treatment. J Biophotonics 11. 10.1002/jbio.201700046 10.1002/jbio.201700046 28700130

[20] Buzzá HH, Zangirolami AC, Kurachi C, Bagnato VS (2019) Acceleration of newborn rats’ development with the use of photobiomodulation and the near possibility of application in human premature babies. J Biophotonics 12:1–6. 10.1002/jbio.201800461 10.1002/jbio.201800461 30972966

[21] Dos Santos Cardoso F, Dos Santos JCC, Gonzalez-Lima F et al (2021) Effects of chronic photobiomodulation with transcranial near-infrared laser on brain metabolomics of young and aged rats. Mol Neurobiol 58:2256–2268. 10.1007/s12035-020-02247-z 33417219 10.1007/s12035-020-02247-z

[22] Cardoso FDS, Barrett DW, Wade Z et al (2022) Photobiomodulation of cytochrome c oxidase by chronic transcranial laser in young and aged brains. Front Neurosci 16:818005. 10.3389/fnins.2022.818005 35368252 10.3389/fnins.2022.818005 PMC8971717

[23] Gutiérrez-Menéndez A, Martínez JA, Méndez M, Arias JL (2022) No effects of photobiomodulation on prefrontal cortex and hippocampal cytochrome C oxidase activity and expression of c-Fos protein of young male and female rats. Front Neurosci 16:897225. 10.3389/fnins.2022.897225 35600629 10.3389/fnins.2022.897225 PMC9120528

[24] Parpura V, Heneka MT, Montana V et al (2012) Glial cells in (patho)physiology. J Neurochem 121:4–27. 10.1111/j.1471-4159.2012.07664.x 22251135 10.1111/j.1471-4159.2012.07664.x PMC3304021

[25] Sofroniew MV, Vinters HV (2010) Astrocytes: biology and pathology. Acta Neuropathol 119:7–35. 10.1007/s00401-009-0619-8 20012068 10.1007/s00401-009-0619-8 PMC2799634

[26] Haim L, Ben, Carrillo-de Sauvage MA, Ceyzériat K, Escartin C (2015) Elusive roles for reactive astrocytes in neurodegenerative diseases. Front Cell Neurosci 9:1–27. 10.3389/fncel.2015.00278 26283915 10.3389/fncel.2015.00278 PMC4522610

[27] Sargsyan SA, Monk PN, Shaw PJ (2005) Microglia as potential contributors to motor neuron injury in amyotrophic lateral sclerosis. Glia 51:241–253. 10.1002/glia.20210 15846792 10.1002/glia.20210

[28] Helmut K, Hanisch UK, Noda M, Verkhratsky A (2011) Physiology of microglia. Physiol Rev 91:461–553. 10.1152/physrev.00011.2010 21527731 10.1152/physrev.00011.2010

[29] Dinarello CA (2000) Proinflammatory cytokines. Chest 118:503–508. 10.1378/chest.118.2.503 10936147 10.1378/chest.118.2.503

[30] Konsman JP, Drukarch B, Van Dam AM (2007) (Peri)vascular production and action of pro-inflammatory cytokines in brain pathology. Clin Sci 112:1–25. 10.1042/CS20060043 10.1042/CS20060043 17132137

[31] Paxinos G, Watson C (2007) The rat brain in stereotaxic coordinates. Sixth Edit. Elsevier

[32] Spijker S (2011) Dissection of rodent brain regions. Neuromethods 57:13–26. 10.1007/978-1-61779-111-6_2

[33] Banqueri M, Méndez M, Gómez-Lázaro E, Arias JL (2019) Early life stress by repeated maternal separation induces long-term neuroinflammatory response in glial cells of male rats. Stress 22:563–570. 10.1080/10253890.2019.1604666 31007117 10.1080/10253890.2019.1604666

[34] Livak KJ, Schmittgen TD (2001) Analysis of relative gene expression data using real-time quantitative PCR and the 2-∆∆CT method. Methods 25:402–408. 10.1006/meth.2001.1262 11846609 10.1006/meth.2001.1262

[35] Blanco NJ, Maddox WT, Gonzalez-Lima F (2017) Improving executive function using transcranial infrared laser stimulation. J Neuropsychol 11:14–25. 10.1111/jnp.12074 26017772 10.1111/jnp.12074 PMC4662930

[36] Hashmi JT, Huang YY, Osmani BZ et al (2010) Role of low-level laser therapy in neurorehabilitation. PM&R 2:S292–S305. 10.1016/j.pmrj.2010.10.013 21172691 10.1016/j.pmrj.2010.10.013 PMC3065857

[37] Liebert A, Bicknell B, Laakso E-L (2021) Improvements in clinical signs of Parkinson’s disease using photobiomodulation: a prospective proof-of-concept study. BMC Neurol 21:256. 10.1186/s12883-021-02248-y 34215216 10.1186/s12883-021-02248-y PMC8249215

[38] O’Donnell CM, Barrett DW, Fink LH et al (2022) Transcranial infrared laser stimulation improves cognition in older bipolar patients: proof of concept study. J Geriatr Psychiatry Neurol 35:321–332. 10.1177/0891988720988906 33525934 10.1177/0891988720988906

[39] Rodríguez-Fernández L, Zorzo C, Arias JL, Springer International Publishing (2024) Photobiomodulation in the aging brain: a systematic review from animal models to humans. GeroScience. 10.1007/s11357-024-01231-y 38861125 10.1007/s11357-024-01231-y PMC11493890

[40] Saltmarche AE, Naeser MA, Ho KF et al (2017) Significant improvement in cognition in mild to moderately severe dementia cases treated with transcranial plus intranasal photobiomodulation: case series report. Photomed Laser Surg 35:432–441. 10.1089/pho.2016.4227 28186867 10.1089/pho.2016.4227 PMC5568598

[41] Moro C, Torres N, Arvanitakis K et al (2017) No evidence for toxicity after long-term photobiomodulation in normal non-human primates. Exp Brain Res 235:3081–3092. 10.1007/s00221-017-5048-7 28744621 10.1007/s00221-017-5048-7

[42] Cassano P, Caldieraro MA, Norton R et al (2019) Reported side effects, weight and blood pressure, after repeated sessions of transcranial photobiomodulation. Photobiomod Photomed Laser Surg 37:651–656. 10.1089/photob.2019.4678 10.1089/photob.2019.4678 31647774

[43] Cassano P, Norton R, Caldieraro MA et al (2022) Tolerability and safety of transcranial photobiomodulation for mood and anxiety disorders. Photonics. 10.3390/photonics9080507

[44] Castaño-Castaño S, Zorzo C, Martínez-Esteban JÁ, Arias JL (2024) Dosimetry in cranial photobiomodulation therapy: effect of cranial thickness and bone density. Lasers Med Sci 39:76. 10.1007/s10103-024-04024-z 38386189 10.1007/s10103-024-04024-z PMC10884051

[45] Kim UJ, Hong N, Ahn JC (2022) Photobiomodulation attenuated cognitive dysfunction and neuroinflammation in a prenatal valproic acid-induced autism spectrum disorder mouse model. Int J Mol Sci. 10.3390/ijms232416099 36555737 10.3390/ijms232416099 PMC9785820

[46] Hamblin MR, Salehpour F (2021) Photobiomodulation of the brain: shining light on Alzheimer’s and other neuropathological diseases. J Alzheimers Dis 83:1395–1397. 10.3233/JAD-210743 34459408 10.3233/JAD-210743

[47] Van Rossum D, Hanisch UK (2004) Microglia. Metab Brain Dis 19:393–411. 10.1023/B:MEBR.0000043984.73063.d8 15554430 10.1023/b:mebr.0000043984.73063.d8

[48] Cardoso FDS, Salehpour F, Coimbra NC (2022) Photobiomodulation for the treatment of neuroinflammation: a systematic review of controlled laboratory animal studies. Front Neurosci 16:1006031 36203812 10.3389/fnins.2022.1006031 PMC9531128

[49] El Massri N, Weinrich TW, Kam JH et al (2018) Photobiomodulation reduces gliosis in the basal ganglia of aged mice. Neurobiol Aging 66:131–137. 10.1016/j.neurobiolaging.2018.02.019 29571001 10.1016/j.neurobiolaging.2018.02.019 PMC5933512

[50] Sanderson TH, Wider JM, Lee I et al (2018) Inhibitory modulation of cytochrome c oxidase activity with specific near-infrared light wavelengths attenuates brain ischemia/reperfusion injury. Sci Rep 8:1–12. 10.1038/s41598-018-21869-x 29472564 10.1038/s41598-018-21869-x PMC5823933

[51] Dompe C, Moncrieff L, Matys J et al (2020) Photobiomodulation—underlying mechanism and clinical applications. J Clin Med 9:1–17. 10.3390/jcm9061724 10.3390/jcm9061724 PMC7356229 32503238

[52] Salehpour F, Rasta SH, Mohaddes G et al (2016) Therapeutic effects of 10-HzPulsed wave lasers in rat depression model: a comparison between near-infrared and red wavelengths. Lasers Surg Med 48:695–705. 10.1002/lsm.22542 27367569 10.1002/lsm.22542

[53] Zorzo C, Fernández LR, Martínez JA, Arias JL (2024) Photobiomodulation increases brain metabolic activity through a combination of 810 and 660 wavelengths: a comparative study in male and female rats. Lasers Med Sci. 10.1007/s10103-023-03966-0 10.1007/s10103-023-03966-0 PMC10786747 38214813

